# Development and characterization of rice bran-gum Arabic based encapsulated biofertilizer for enhanced shelf life and controlled bacterial release

**DOI:** 10.3389/fmicb.2023.1267730

**Published:** 2023-09-19

**Authors:** Rajinder Kaur, Sukhminderjit Kaur, Vagish Dwibedi, Charanjit Kaur, Nadeem Akhtar, Abdulhakeem Alzahrani

**Affiliations:** ^1^Department of Biotechnology, University Institute of Biotechnology, Chandigarh University, Mohali, India; ^2^Department of Microbiology, Bhojia Institute of Life Sciences, Baddi, India; ^3^Department of Animal Biosciences, University of Guelph, Guelph, ON, Canada; ^4^Department of Food Science & Nutrition, College of Food and Agricultural Sciences, King Saud University, Riyadh, Saudi Arabia

**Keywords:** biofertilizer, carrier-based biofertilizers, encapsulation, liquid biofertilizer, *Myroid gitamensis*, plant growth promotion

## Abstract

**Introduction:**

Currently, microbe-based approaches are being tested to address nutrient deficiencies and enhance nutrient use efficiency in crops. However, these bioinoculants have been unsuccessful at the commercial level due to differences in field and *in-vivo* conditions. Thus, to enhance bacterial stability, microbial formulations are considered, which will provide an appropriate microenvironment and protection to the bacteria ensuring better rhizospheric-colonization.

**Methods:**

The present study aimed to develop a phosphobacterium-based encapsulated biofertilizer using the ion-chelation method, wherein a bacterial strain, *Myroid gitamensis* was mixed with a composite solution containing rice bran (RB), gum Arabic (GA), tricalcium phosphate, and alginate to develop low-cost and slow-release microbeads. The developed microbead was studied for encapsulation efficiency, shape, size, external morphology, shelf-life, soil release behavior, and biodegradability and characterized using SEM, FTIR, and XRD. Further, the wheat growth-promoting potential of microbeads was studied.

**Results:**

The developed microbeads showed an encapsulation efficiency of 94.11%. The air-dried beads stored at 4°C were favorable for bacterial survival for upto 6 months. Microbeads showed 99.75% degradation within 110 days of incubation showing the bio-sustainable nature of the beads. The application of dried formulations to the pot-grown wheat seedlings resulted in a higher germination rate, shoot length, root length, fresh weight, dry weight of the seedlings, and higher potassium and phosphorus uptake in wheat.

**Discussion:**

This study, for the first time, provides evidence that compared to liquid biofertilizers, the RB-GA encapsulated bacteria have better potential of enhancing wheat growth and can be foreseen as a future fertilizer option for wheat.

## 1. Introduction

Microbial biofertilizers are a type of fertilizer that contains living microorganisms, such as bacteria, fungi, and algae, which can promote plant growth and health. These microorganisms can interact with the plant root system and soil to provide essential nutrients, fix atmospheric nitrogen, increase nutrient availability, and enhance soil fertility (Fasusi et al., 2021; Kaur et al., 2021). The use of microbial biofertilizers as a partial replacement to chemical fertilizers has gained increasing attention in recent years due to their environmentally friendly nature, low cost, and potential to improve soil health and plant productivity. Moreover, microbial biofertilizers have been found to reduce the environmental impact of agriculture, promote sustainable farming practices, and increase crop yields. Different types of microorganisms can be used as biofertilizers, depending on the specific needs of the plant and soil (Kaur and Kaur, 2021). For example, nitrogen-fixing bacteria like *Rhizobium* and *Azotobacter* can convert atmospheric nitrogen into a form that plants can use, while phosphate-solubilizing bacteria like *Pseudomonas* and *Bacillus* can increase the availability of phosphorus in the soil (Ahemad and Kibret, 2014; Kaur and Kaur, 2018; Bargaz et al., 2021; Rani et al., 2021).

The direct application of these microbes into the soil causes them to have a tough time growing and colonizing the plant roots. The viability and functionality of the biofertilizer are adversely affected (Shah et al., 2021) due to high contamination, low shelf life, and competition with the rhizospheric microbe. The low acceptance of biofertilizers among farmers corresponds to poor performance in the field conditions. Therefore, to boost the resilience and viability of the microbes for harnessing their desired potential of plant growth promotion, bioformulation is prepared using selecting and culturing specific strains of microorganisms, combining them with carriers, and formulating them into a product that can be easily applied to plants and soil. The goal of microbial bioformulation is to create a product that can deliver a specific type of microorganism to the plant and soil in a stable and effective manner. The carrier materials used in bioformulation can range from organic materials like peat, vermiculite, and compost to synthetic materials like hydrogels, polymers, and nanomaterials (Aloo et al., 2022). Even though many different types of liquid and solid/granular formulations are available in the market; the commercial acceptability of these formulations among farmers is very less. Solid formulations include powder or granular formulations made by mixing microbial culture with cornstarch, gum acacia, gluten, wheat granules, molasses, charcoal, farm manure, fly ash, or wheat flour (Fathi et al., 2021; Meftah Kadmiri et al., 2021).

The use of microencapsulation technology in biofertilizer production offers several advantages over traditional methods. Microencapsulation is the process of enclosing a material or substance within a small capsule or sphere, often made of a biodegradable polymer, to protect it from environmental factors and control its release. The microencapsulated biofertilizer can be applied directly to the soil, reducing the need for frequent applications, and preventing leaching into the surrounding environment. The slow-release behavior of the biofertilizer ensures that nutrients are available to the plant for a longer period of time, resulting in improved plant growth, increased crop yields, and reduced nutrient runoff (Rivett et al., 2018; Tosi et al., 2020). Additionally, the slow-release behavior of the biofertilizer can reduce the frequency of application, resulting in cost savings for farmers and gardeners.

The encapsulated biofertilizer are made using cheap and affordable raw materials, which helps keep the microbial cells viable (Razavi et al., 2021). The matrix in the process is made up of biodegradable and non-toxic alginate, and gum Arabic. Gum Arabic provides excellent stability and protection to the microcapsules and acts as a prebiotic, promoting the growth of beneficial bacteria in the soil (He et al., 2015, 2017). To provide protection against high temperatures and provide a constant supply of nutrients to microbes, rice bran is used as a filler material (Zhu, 2007; Katz et al., 2015). The interaction of alginate and gum Arabic with rice bran provides a non-porous dense structure to the bead. This dense layer is useful for the success of the post-application of microbeads in the soil. As this outer layer dissolute, it may gradually release bacteria continuously into the soil in a slow gradual manner (Meftah Kadmiri et al., 2021).

It is uncertain how the encapsulated biofertilizers behave in soil. The present study was carried out with the objective of developing a biodegradable microencapsulated biofertilizer that can have a high shelf life, low contamination rate, and slow-release behavior in soil. To study the shelf-life, contamination rate, and release behavior of encapsulated biofertilizers, phosphobacterium *Myroid gitamensis* (GenBank Accession no. MT835236) which was previously isolated from the uncultivated soils of Punjab, was encapsulated in alginate-gum Arabic matrix. Rice bran was used as a natural filler to decrease porosity and provide heat protection. The encapsulation efficiency, morphology, release kinetics, and biodegradability behavior of the developed microbead were studied.

## 2. Materials and methods

### 2.1. Sub-culturing and cell culture preparation

“*Myroid gitamensis*” BSH-3 a strain previously isolated from the soil sample collected from uncultivated land of the Mohali district of Punjab, India (30°53′ N 76°38′ E) (Kaur and Kaur, 2021) was used throughout the study. The pure culture was grown in Pikovskaya agar medium supplemented with 5% tricalcium phosphate (w/v) and 5% soya lecithin (w/v). The purified culture was preserved in glycerol stock at −20°C. The cells were sub-cultured in 5 mL nutrient broth (HiMedia) at 28 ± 2°C for 24 h. Then, the cultures were transferred into 500 mL of nutrient broth and incubated to grow in continuous shaking conditions at 28°C until the bacterial strain reached a concentration of 10^12^CFU/mL (Kumar et al., 2017). Cell pellets were harvested by centrifugation at 10,000*g* for 10 min at 4°C (Remi Centrifuge VCBS2252, Vasai, India). The pellets were washed three times with sterile 0.5 M phosphate buffer and suspended in 1 mL sterile distilled water to obtain concentrated “*M. gitamensis*” BSH-3 (~10^9^ CFU/mL) (Kaur and Kaur, 2023).

### 2.2. Encapsulation of *Myroid gitamensis* in RB-GA microbeads

**Figure 1:**
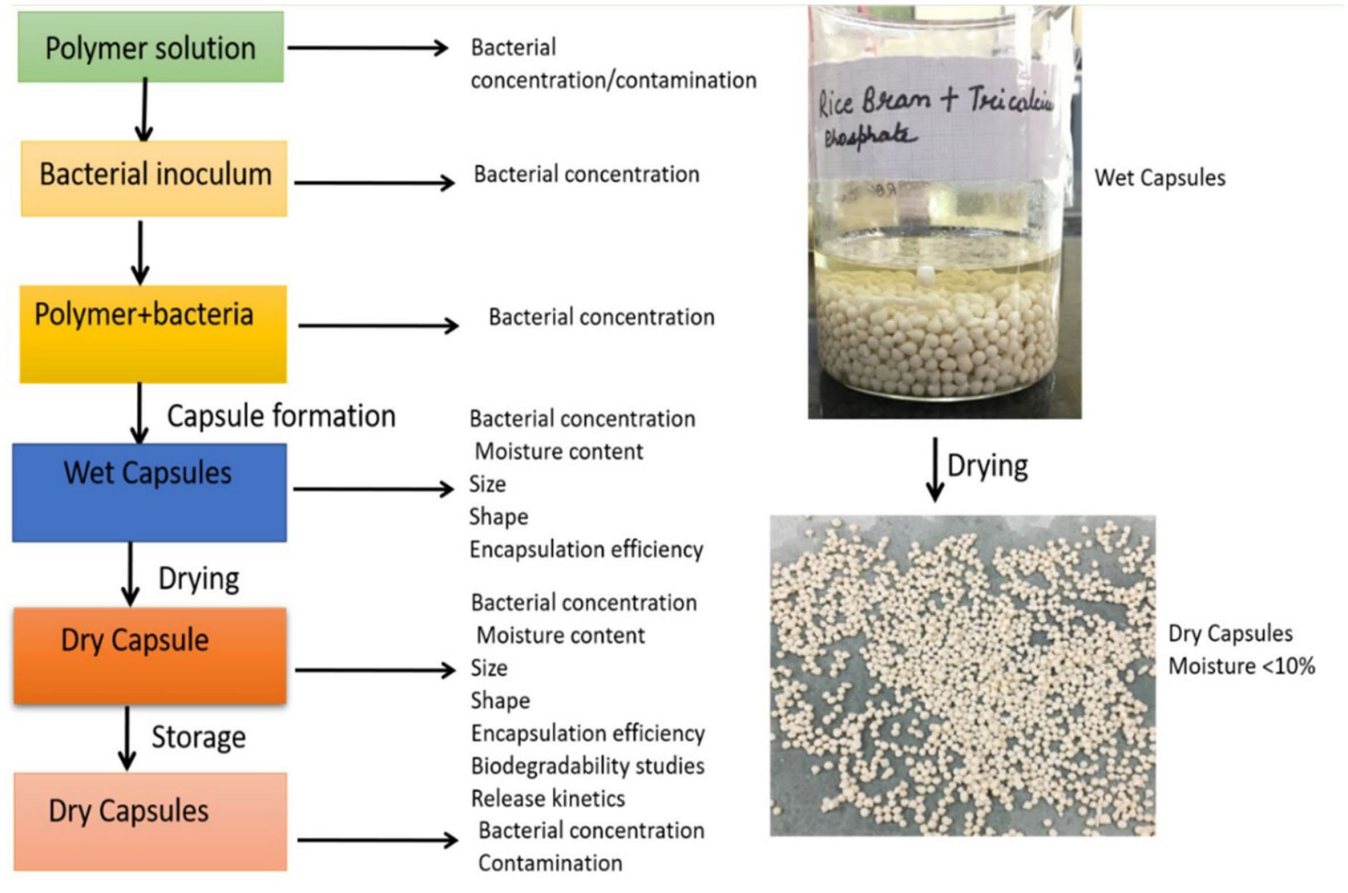
Analysis of developed encapsulated biofertilizer at different encapsulation steps.

A polymeric solution is prepared by mixing 2% (w/v) of sodium alginate and 2% (w/v) gum Arabic in hot water followed by the addition of 2.5% (w/v) rice bran, 2.5% w/v of tricalcium phosphate, 5% glycerol (analytical grade) and 0.1% by volume of tween 20. The bacterial cell pellet and the polymeric solution are mixed to form a uniform suspension. This suspension is continuously shaken on an orbital shaker for 1 h at 28°C. The colony forming units (CFU/mL) in the polymer suspension was measured using the spread plate technique using Pikovskaya agar containing 5% tricalcium phosphate and 5% soya lecithin and incubating at 28°C at 1 h. The uniform suspension is then added dropwise to 0.5 M calcium chloride solution to form microbeads. The microbeads were allowed to harden in a calcium chloride solution over-night. The microbeads are taken out of the calcium chloride solution and washed twice with sterile distilled water. The beads were air-dried at room temperature and dried in an oven at 37 ± 2°C to a moisture content below 10% and stored in a sterile container (Kaur and Kaur, 2023). The developed bead was analyzed through a detailed methodology briefly described in Figure 1.

### 2.3. Enumeration of viable cells in RB-GA microbeads and encapsulation efficiency

The viable cell count was done by an improved pour plate method (Terrones-Fernandez et al., 2023). The 72 h old dried beads (10 beads) were rehydrated using 0.8% NaCl solution (w/w, pH-7). The rehydrated beads were derecticulated by suspending in 10 mL sodium-tricitrate (10% w/w) followed by shaking for 30 min. The suspension obtained was serially diluted and 10 μL of the dilution was spread on the Pikovskaya agar plates for bacterial enumeration (Petraitytė and Šipailienė, 2019). The bacterial cell count was measured after incubating the plates at 28°C for 24 h and expressed as colony-forming units per gram. The encapsulation efficiency was calculated using the following formula:


$$\mathrm{EE}\left(\%\right)=\frac{N_{0}-N}{N_{0}}\times 100.$$

*N* is the number of cells released from the beads after 24 h (log CFU/g of bead), *N*_0_ is the number of cells in the polymer solution at the time of encapsulation (log CFU/g of polymer solution).

### 2.4. Shape and size of dried microbeads

The size of 10 dry beads was measured with a stereomicroscope microscope (Olympus) and size was quantified as sphericity factor by the given formula:


$$\mathrm{sphericity}\;\;\mathrm{factor}\;\;\left(\mathrm{SF}\right)=\frac{d_{\max}-d_{\min}}{d_{\max}+d_{\min}}.$$

where *d*_max_ is the largest diameter of beads and *d*_min_ is the smallest diameter of the bead perpendicular to *d*_max_.

### 2.5. Storage studies

The wet and dried beads (72 h old beads) were stored in sterile glass vials at room temperature and at 4°C. The shelf life of the beads was studied by rehydrating the dry beads and derecticulating, followed by quantification of viable cell count by pour plate method (Liffourrena and Lucchesi, 2018). The storage studies were done after every 15 days up to 180 days. All the data was presented in Mean ± SD of triplicate and the statistical significance of data was evaluated according to Tukey’s post-hoc analysis (*p* < 0.05).

### 2.6. Behavior of beads in soil/biodegradability

Through the observation of mass loss during the incubation span of 1 month in an open field environment, the rate of biodegradation of the bead was evaluated (He et al., 2015). Therefore, 10 mg (*W*_i_) dried beads were stowed in a nylon packet and buried about 5 cm below the soil surface. To soil, 10 mL water was added every day and the open field conditions were maintained. The calculation of weight loss at various time intervals (*W*_d_) as well as the measurement of the rate of biodegradation was determined in accordance with the below-mentioned formula. All the experiments were performed thrice.


$$\mathrm{Biodegradation}=\frac{W_{i}-W_{d}}{W_{d}}\times 10.$$

### 2.7. Release behavior of the bacterial cells from the microbeads

To study the release behavior of beads, 1 g of the beads was dipped in 10 mL of sterile saline solution and kept for 30 days at room temperature. At different time periods, aliquots of 100 μL were removed. The nutrient agar plate count method was employed for the determination of the number of live cells in the solution. Results were exhibited as the release of viable cells Log CFU g^−1^ (He et al., 2015).

### 2.8. Characterization of microbeads

#### 2.8.1. Fourier transform infrared spectrometry (FTIR)

To analyze the structure of the microbead and to estimate the presence of different bonds in the polymer matrix, FTIR analysis was carried out using an FTIR spectrometer. The beads were vacuum dried and ground into powder and mixed with potassium bromide powder in a ratio of 1:200. The powder is then compressed to make a disk for FTIR characterization. The spectra were collected at 4 mm/s at 2 cm^−1^ resolution over the range from 4,000 to 400 cm^−1^ with 50 scans and a resolution of 4 cm^−1^. The characteristic peaks at wavenumber were recorded and checked for characteristic functional groups present in molecular structure.

#### 2.8.2. X-ray diffraction (XRD) analysis

By using Cu as anode material at room temperature, an XPERT-PRO X-ray diffractometer was employed for the measurement of small-angle X-ray Reflection. Into an aluminum holder, samples were packed and the diffractograms were recorded in the 2q angle range from 5.0084° to 49.9904° with the following operating parameter settings: scan step size at 0.0170° (2q), scan step time at 24.765 s (Zheng and Chow, 2009).

#### 2.8.3. Scanning electron microscopy to characterize the surface morphology of microbeads

A scanning electron microscope (JSM-IT 500, JEOL) was used to study the internal and external surfaces of the microbeads. The beads were incubated in osmium tetroxide solution (2%) for 1 h and then washed with distilled water for 10 min. The microbeads were dehydrated in 30% ethanol (v/v) for 10 min followed by dehydration in 50, 70 and 90%, and 100% ethanol solution for 10 min. Afterwards, using critical point-drying they were dried under vacuum. Using double-sided adherent tape, microbeads were attached to aluminum stubs and coated with gold using ion sputtering coaters (Chitprasert et al., 2012).

### 2.9. Effect of RB-GA microencapsulated *Myroid gitamensis* biofertilizer application on wheat seedlings

The effect of RB-GA microencapsulated *M. gitamensis* on wheat seedlings was studied by conducting a pot experiment. Wheat (*T. aestivum*) seeds of the local variety PBW 803 were surface sterilized by dipping in 0.1% mercuric chloride followed by repeated rinsing in distilled water. According to the recommended package of practices, PBW 803 takes 151 days to mature and requires 55 kg/acre of diammonium potassium and 40 kg/acre of muriate of potash to grow, indicating that it is a high nutrient-requiring crop.^1^ Sterile seeds were soaked overnight in autoclaved distilled water. Autoclaved soil was filled in 12.5 cm pots and 20 seeds were sown in each pot. For the test 2 g of RB-GA encapsulated bacteria were added to the soil at the time of sowing. For positive control, 2 g of vermicompost was added to the pot while autoclaved RB-GA beads served as negative control. Also, in another pot, 2 mL of 24 h old liquid culture grown in nutrient broth was added. Pots were incubated in open conditions during the winter season to mimic the natural conditions and water every 48 h. The germination rate, shoot length, root length, fresh weight, and dry weight of the seedlings were measured after 90 days from the date of sowing. The fresh biomass was evaluated for available nitrogen, potassium, and phosphorus content by the standard procedures reported by Jackson (1973).

### 2.10. Statistical analysis

The data was presented in Mean ± SD of triplicate and the statistical significance of data was evaluated according to Tukey’s post-hoc analysis (*p* < 0.05). The Pearson correlation coefficient was used to study the relationship between mineral uptake and plant growth parameters.

## 3. Results

### 3.1. Encapsulation efficiency, shape, and size of beads

The viable cell count in the beads was determined at each step of encapsulation. *M. gitamensis’* BSH-3 cell concentration approx. of 1 × 10^9^ CFU/mL was added to the polymer solution. The bacterial cell count increased to 31 × 10^9^ CFU/mL after 1 h of incubation. The bacterial count in the hardened encapsulated beads was found to be 15 × 10^9^ CFU/mL. This corresponds to the excellent encapsulation efficiency (EE) of 94.11%. As determined by calculating the sphericity factor (SF), the beads were found to be spherical in shape. The sphericity factor of the bead was found to be 0.2. The dried beads had the largest diameter of 1.23 ± 0.10 mm.

### 3.2. Storage studies

**Figure 2:**
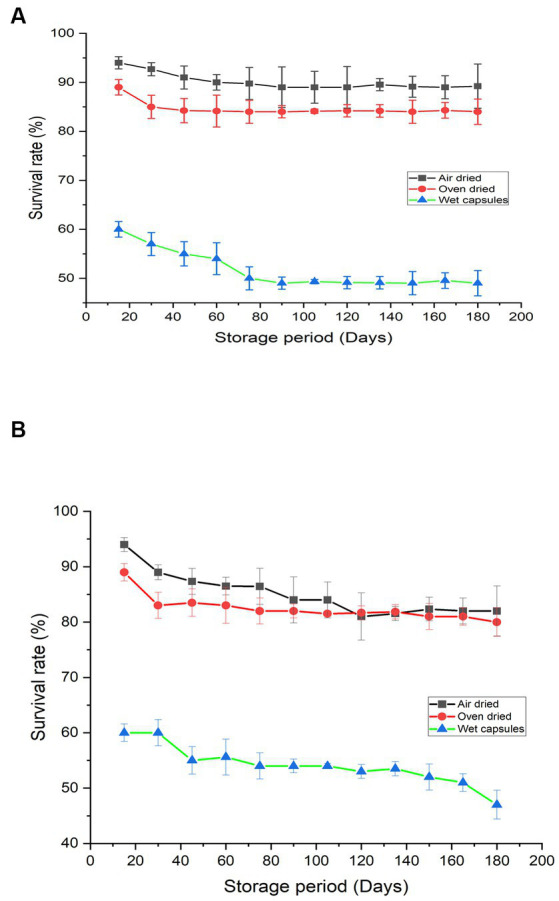
Survival percentage of “*M.gitamensis*” in RB-GA beads stored at **(A)** 4°C **(B)** at room temperature, At *p* < 0.05, the mean survival rate of bacteria in air dried, oven dried and wet capsules varied significantly.

Encapsulated beads were stored at room temperature and 4°C to analyze the survival of “*M. gitamenis*” for 180 days. The viable cell count in the encapsulated bead decreased during the initial phase of storage (Figure 2A). Compared to the wet beads in which the survival rate was observed to be 60%, the air-dried beads (dried at room temperature) showed a viable cell count of 94% while the oven-dried (40°C) showed a survival rate of 89% (Figure 2A). After the initial decrease in the survival rate, the viable cell count remained almost constant for 180 days (Figure 2A).

Compared to the beads stored at room temperature, the beads stored at 4°C showed a higher survival rate (Figure 2B). Also, it is important to note that dried beads were free from contamination throughout the storage period, while the wet beads (both stored at room temperature and at 4°C) were prone to bacterial and fungal contamination over the long storage periods. Therefore, the air-dried beads stored at 4°C were favorable for bacterial survival over the long storage hours.

### 3.3. Biodegradability studies and release kinetics

**Figure 3:**
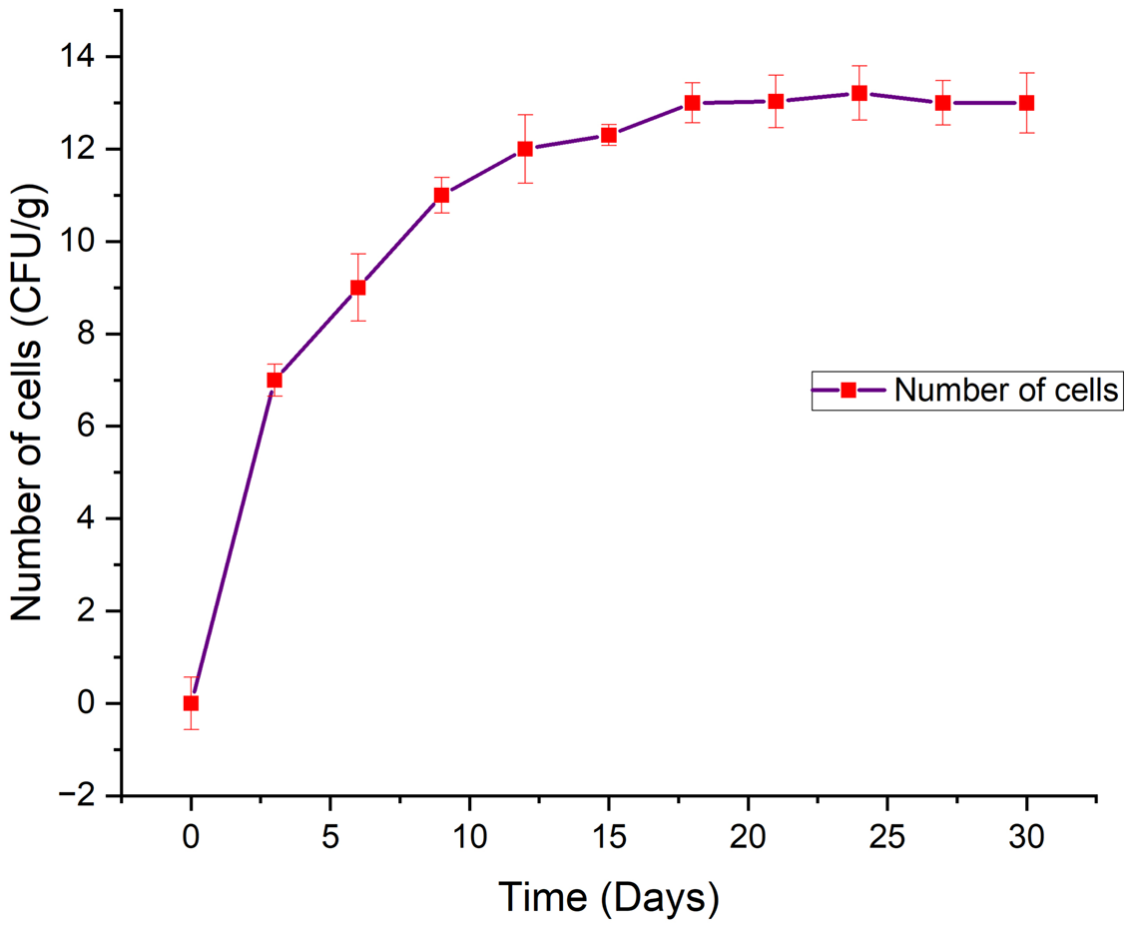
Biodegradation percentage of RB-GA beads at different time interval.

**Figure 4:**
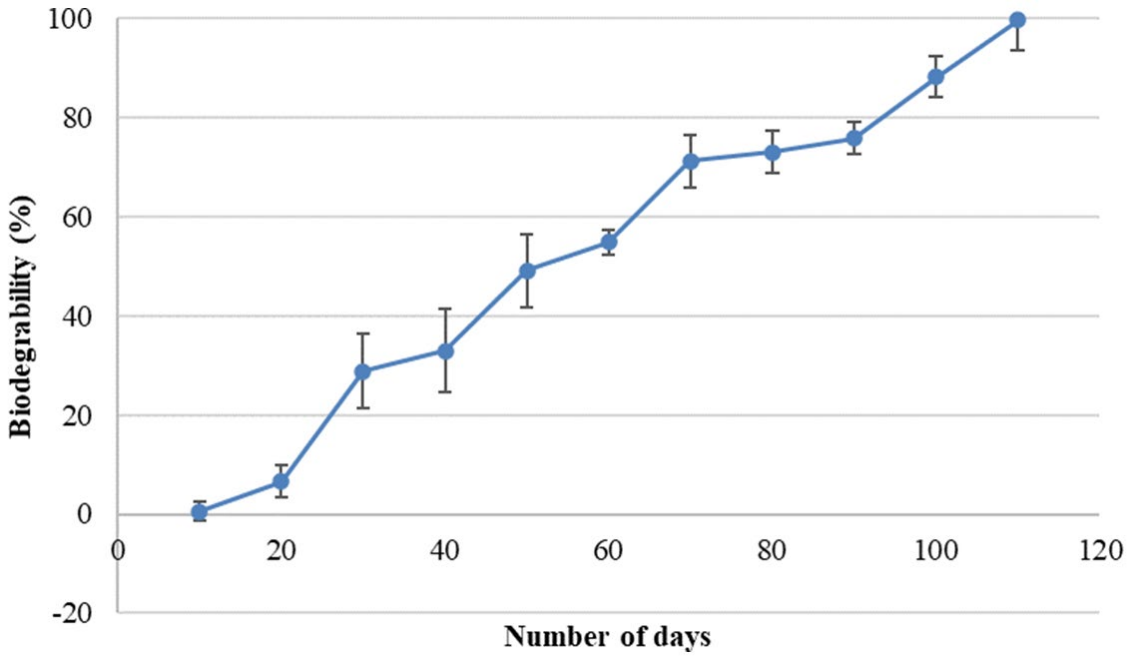
Release pattern of bacterial cells from the RB-GA beads.

In the present study, a biodegradability rate corresponding to 99.75% was observed in 110 days of incubation (Figure 3). Approximately 50% of the bead was disintegrated in 50 days. The rate of degradation of beads in natural soil conditions was steady. It took approximately 4 months for the bead to completely disintegrate into the soil confirming the slow-release behavior of the bead. The rate of bead degradation was found to be significantly negatively correlated to the bead size (r = −0.93). Similar to the observation made by He et al. (2015) The release of bacterial cells from the bead was observed in two phases, initial burst followed by steady and continuous release (Figure 4). In the initial phase, between 0 and 3 days, living cells were released rapidly and reached up to 10^7^ cfu/g. From 3 to 9 days, bacterial release slowed down and was gradual reaching up to 10^11^ cfu/g. After 18 days, the bacterial cell release becomes steady with approximate bacterial cell concentration up to 10^13^ cfu/g.

### 3.4. Characterization of microbeads

**Figure 5:**
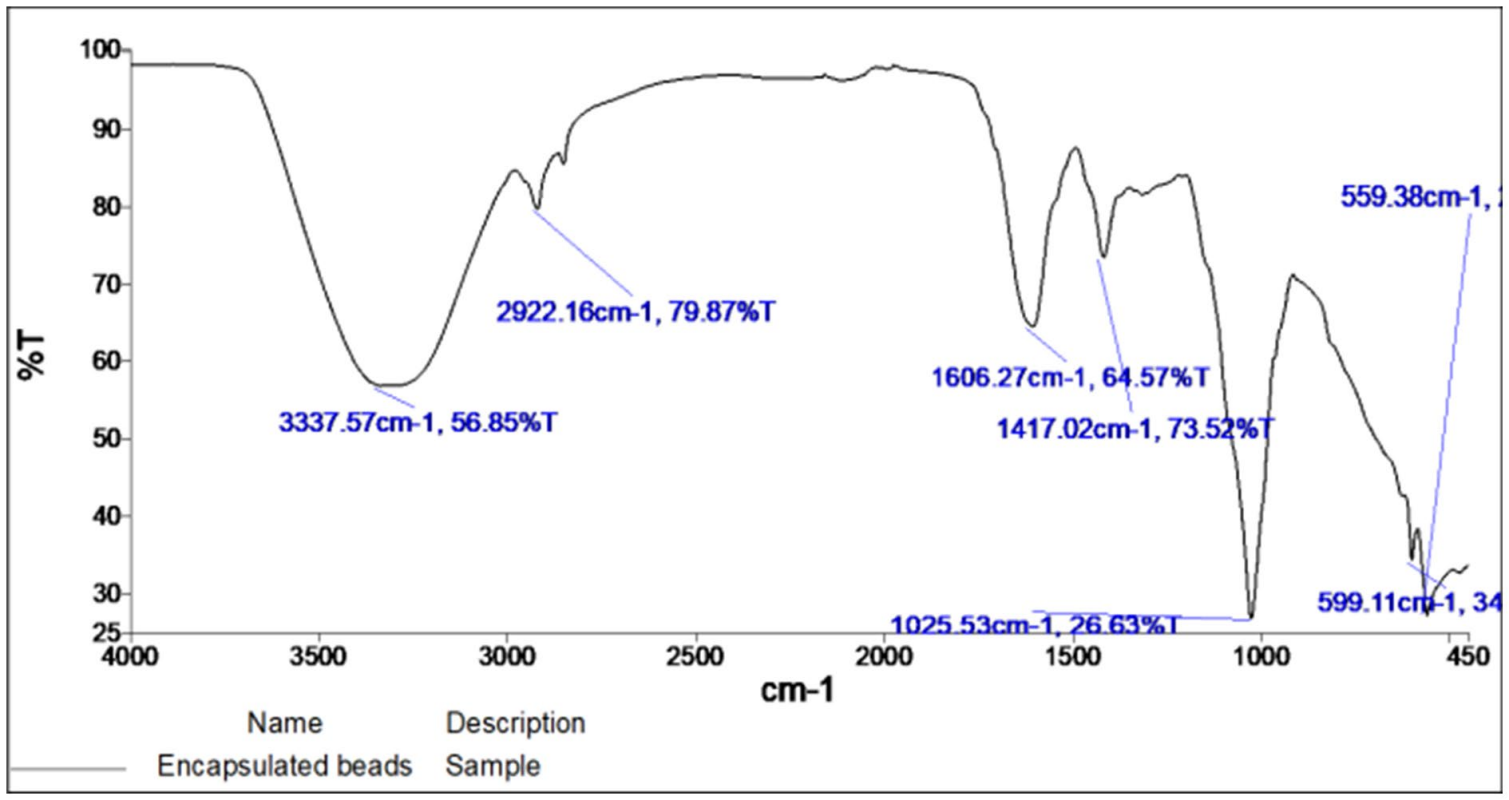
FT-IR spectra of encapsulated beads.

The FTIR spectra of encapsulated beads are shown in Figure 5. The principal IR absorption peaks of the bead exhibited the characteristic peaks associated with the constituents present in it. In the functional group region of the FTIR spectrum, principal IR absorption peaks were witnessed at 3,337.57 cm^−1^ confirming the O-H stretching and sp3 C–H stretching in glucose. Because of the bioactive compounds in rice bran, the characteristic peak was observed at 2,922.16 cm^−1^, confirming the presence the methylene groups (CH_2_). Additionally, the bands at 1,606.27 cm^−1^, 1,417.02 cm^−1^ correspond to the asymmetric stretching vibration of COO groups and the symmetric stretching vibration of COO groups, respectively. Peak at a low wave number of 1,025.53 cm^−1^ represents the stretching mode of PO_4_^3−^ group.

While peaks at 599.11 cm^−1^ and 559.38 cm^−1^ confirmed the vibration peaks of PO_4_^3−^ in β-TCP. These observed principal peaks confirmed the presence and authenticity of the tricalcium phosphate, gum Arabic, sodium alginate, and rice bran (Table 1).

**Table 1:** FTIR interpretation of encapsulated beads.

S. No.	Functional group	Reported (cm^−1^)	Observed (cm^−1^)
1.	O-H Stretching, characteristic to glycosidic linkage	3,313.32	3,337.57
2.	Presence of methylene groups (CH2)	2,930.00	2,922.16
3.	Asymmetric stretching vibration of COO groups	1,635.00	1,606.27
4.	Symmetric stretching vibration of COO groups	1,419.00	1,417.02
5.	Stretching mode of PO4^3−^ group	1,225–950	1,025.53
6.	Vibration peaks of PO4^3−^ in β-TCP	607.00	599.11
		561.00	559.38

**Figure 6:**
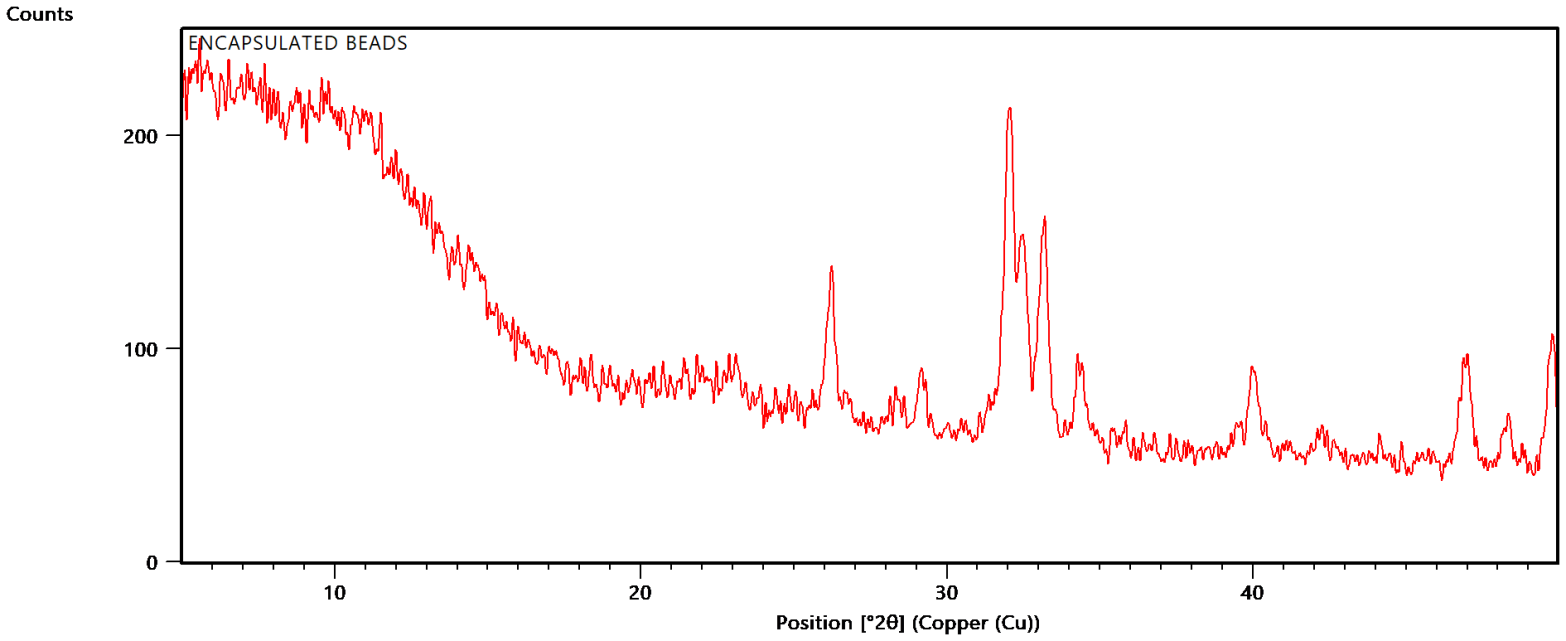
XRD spectra of encapsulated beads.

XRD analysis was carried out to relate the main minerals, associated impurities, and crystalline phases of native organic polymers. The XRD analysis of encapsulated beads at 2θ position showed sharp peaks at 26.188, 29.149, 32.006, 32.463, 33.136, 34.326, 39.979, 42.205, 46.909, and 48.269 owing to partial crystalline nature of formulation (Figure 6). However, a shift was witnessed in the characteristic peak directing towards the influence of the excipient due to encapsulation. As far as sodium alginate is concerned, the characteristic peak was witnessed at 34.326, 39.379, demonstrating its semi-crystalline nature.

**Figure 7:**
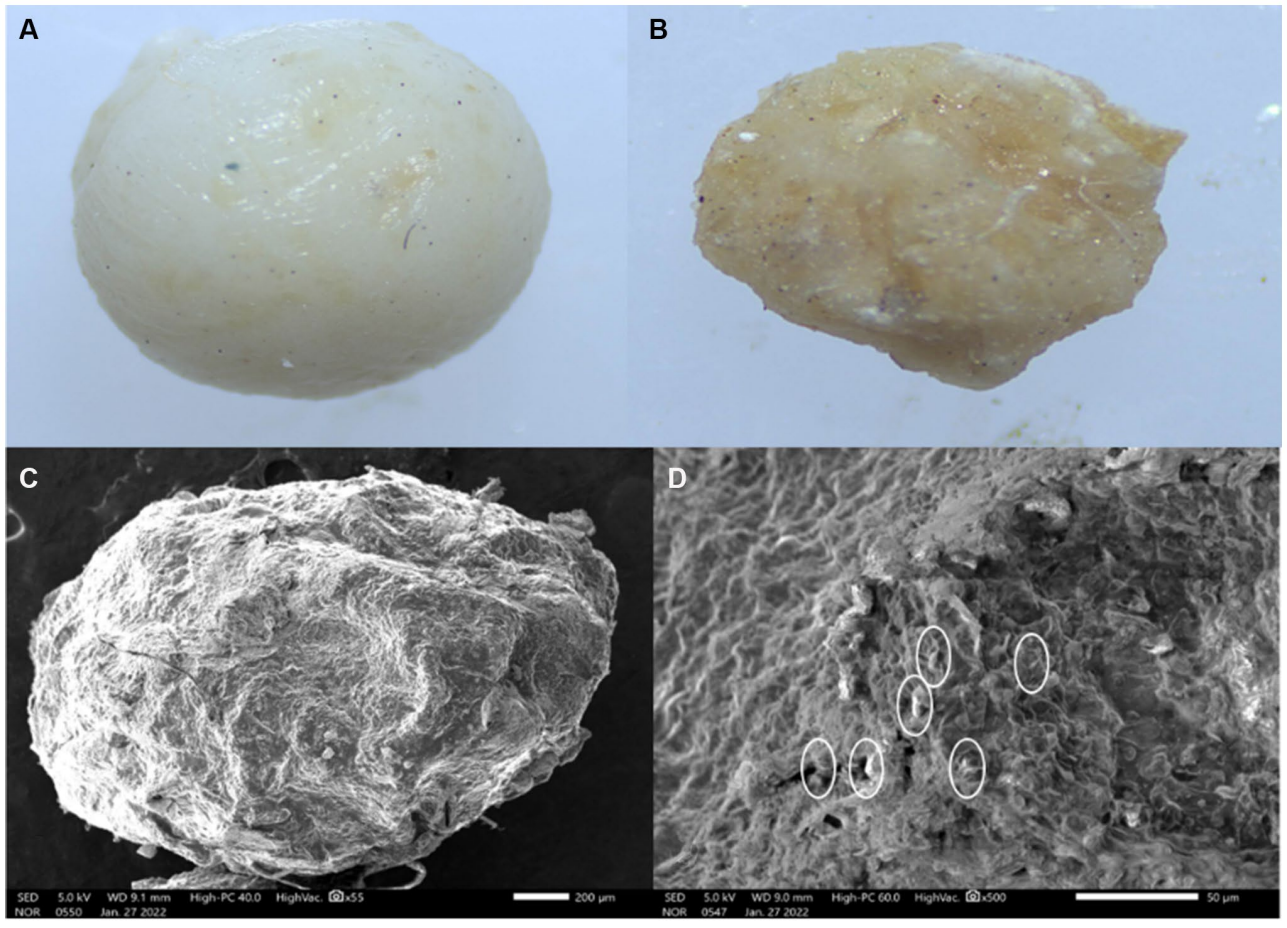
External morphology of encapsulated bead. **(A)** Stereomicroscopic image of wet bead. **(B)** Stereomicroscopic image of dried bead. **(C)** External morphology of bead under scanning electron microscope (SEM) at low magnification 1,200 × 40,000. **(D)** External morphology of bead under SEM at high magnification 2,400 × 40,000.

The external morphology of air-dried beads was studied using a scanning electron microscope as shown in Figure 7A. Beads had rough surfaces and no visible cracks were seen as illustrated in Figure 7D. The exterior of the bead was dense matrices which is essential for protection against heat and inhibition of water penetration. The bacterial cells were not observed on the surface of the bead but were found to be randomly entrapped in the overlaps between rice bran and alginate-gum Arabic matrix (as shown by small circles in Figure 7D) and alginate-gum Arabic matrix.

### 3.5. Effect of biofertilizer on wheat

**Figure 8:**
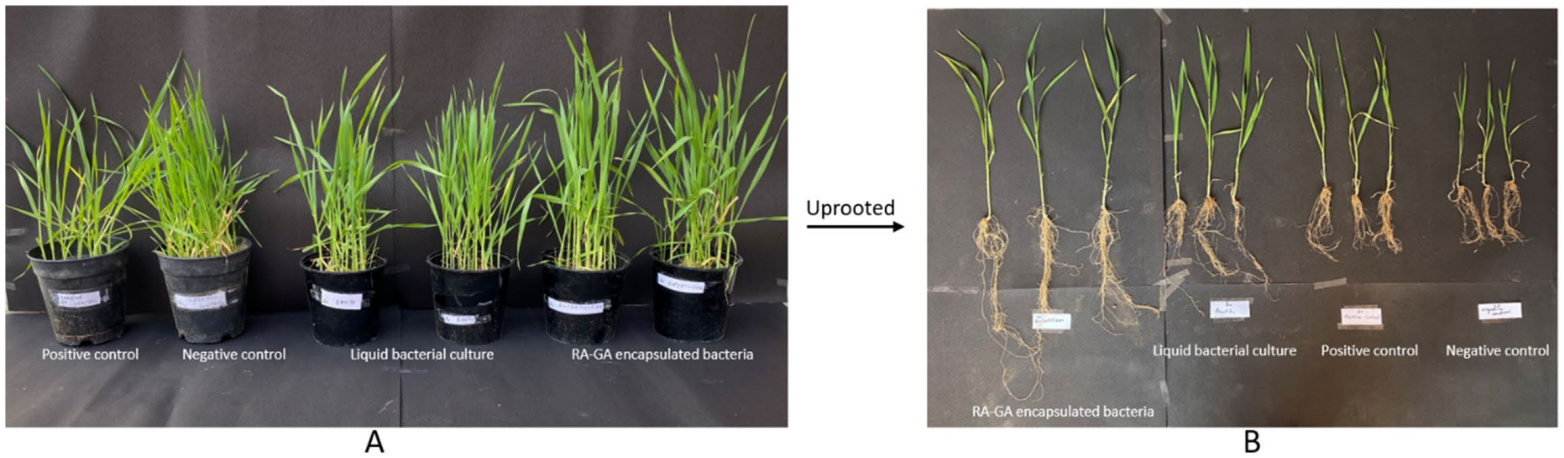
**(A)** Growth of Wheat seedlings in positive and negative control, liquid bacterial culture and RA-GA encapsulated bacterial treated pots after 90 days of sowing. **(B)** Uprooted seedlings from positive and negative control, liquid bacterial culture and RA-GA encapsulated bacterial treated pots after 90 days of sowing in test and control after 90 days.

The plant growth-promoting ability of RB-GA encapsulated *M. gitamensis* was assessed by supplying the dried bioformulations to the pot-grown wheat seedlings allowed to grow in natural open field conditions. Table 2 represents the effect of RB-GA bioformulation on nutrient uptake, plant growth, and biomass. The germination rate of 97.16% was found in the RB-GA-treated pot which was significantly higher than positive control (89.66%) and negative control (81.66%). Visible differences were found in the shoot length with the highest shoot length of 38.33 cm observed in RB-GA-treated pots, 35.5 cm in broth, 33.66 cm in positive, and 24.66 cm in negative control (Figure 8, Table 2). Root length was also found to be the highest (41 cm) in root length, 27 cm in broth, 15.33 cm in positive control, and 14.83 cm in negative control. Compared to negative and positive controls, root length, fresh weight, and dry weight of the seedlings were significantly higher in the RB-GA-treated pots. Wheat seedling inoculants with beads showed the highest fresh weight and dry weight of 24.94 g and 18.70 g, respectively, which was statistically significantly higher than other treatments.

**Table 2:** Plant growth promotion of RB-GA encapsulated *M. gitemensis.*

Parameters	RB-GA beads	Bacterial culture in nutrient broth	Positive control	Negative control
Germination rate (%)	97.16 ± 1.04^a^	94.83 ± 0.76^a^	89.66 ± 0.57^b^	81.66 ± 1.52^c^
Shoot length (cm)	38.33 ± 1.52^a^	35.5 ± 1.80^ab^	33.66 ± 2.31^b^	24.66 ± 0.57^c^
Root length (cm)	41 ± 2.64^a^	27 ± 1.73^b^	15.33 ± 3.05^c^	14.83 ± 0.76^c^
Fresh weight (g)	24.94 ± 0.41^a^	20 ± 0.98^b^	17.16 ± 0.28^c^	13.83 ± 0.76^d^
Dry weight (g)	18.70 ± 0.37^a^	14.42 ± 1.12^b^	12.10 ± 0.63^c^	12.12 ± 0.63^d^
Nitrogen content (mg/100 g)	11.12 ± 0.76^a^	11.23 ± 0.65^b^	15.73 ± 0.61^c^	10.5 ± 0.32^d^
Potassium content (mg/100 g)	9.24 ± 0.24^a^	7.10 ± 0.22^b^	4.05 ± 0.45^c^	1.32 ± 0.66^d^
Phosphorus content (mg/100 g)	60.10 ± 0.97^a^	54.50 ± 0.14^b^	48.72 ± 0.98^c^	37.51 ± 0.10^d^

Data is represented as Mean ± S.D of three independent replicates. Means that do not share the same letter are significantly different (*p* < 0.05).

**Figure 9:**
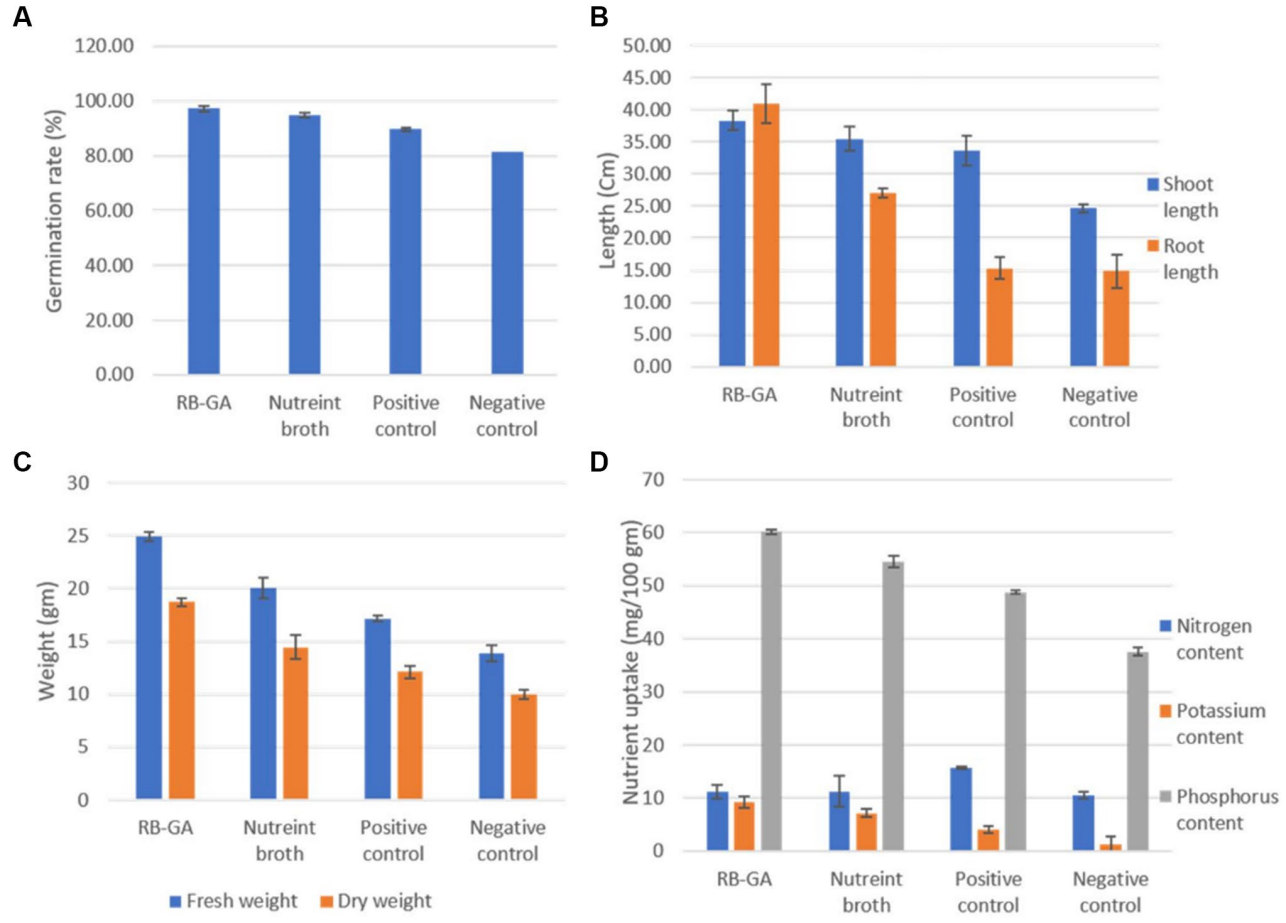
Comparative analysis of effect of RB-GA encapsulated *M. gitamensis* and broth culture on **(A)** Germination rate. **(B)** Shoot and root length. **(C)** Fresh and dry weight. (D) Nutrient uptake.

Also, the bacteria were reported to have its inheritant effective phosphate-solubilizing (PS), and potassium solubilizing (KS) phosphate-mineralizing (PM) potential. Encapsulated beads showed significantly higher potassium (9.24 mg/100 g) and phosphorus (60.10 mg/100) uptake in wheat compared to broth-inoculated wheat which shows 7.10 mg/100 g potassium and 54.50 mg/100 phosphorus uptake. Nitrogen content in RB-GA treated and broth treated was found to be 11.12 mg/100 g and 11.23 mg/100 g, respectively. No significant difference was observed in the nitrogen uptake in the RB-GA bead-treated pots (Figure 9). The Pearson correlation test was used to observe the relationship between mineral uptake and wheat growth parameters. Phosphorus content was found to be highly positively correlated to the root length, shoot length, fresh weight, and dry weight with r values of 0.87, 0.98, 0.96, and 0.83, respectively. This indicates that phosphorus is an important factor in determining wheat biomass and growth. Similarly, potassium content was also found to be highly positively correlated to the root length, shoot length, fresh weight, and dry weight with r values of 0.93, 0.94, 0.98, and 0.90, respectively. A negligible correlation was found between nitrogen content and the wheat growth parameters (Table 3).

**Table 3:** The Pearson correlation matrix between mineral uptake and wheat growth parameters.

	Root length	Shoot length	Fresh weight	Dry weight	Germination rate	Phosphorus content	Nitrogen content	Potassium content
Root Length	1							
Shoot Length	0.77	1						
Fresh weight	0.96	0.91	1.00					
Dry weight	0.99	0.74	0.95	1				
Germination rate	0.84	0.98	0.94	0.79	1.00			
Phosphorus content	**0.87** ^a^	**0.98** ^a^	**0.96** ^a^	**0.83** ^a^	0.99	1.00		
Nitrogen content	−0.41	0.20	−0.15	−0.40	0.01	0.02	1.00	
Potassium content	**0.93** ^a^	**0.94** ^a^	**0.98** ^a^	**0.90** ^a^	0.98	0.99	−0.14	1.00

a Represents high positive correlation.

## 4. Discussion

With the aim of developing a biodegradable microencapsulated biofertilizer phosphobacterium *Myroid gitamensis* was encapsulated in alginate-gum Arabic matrix. In the present study, the sphericity factor of the bead was found to be 0.2. The sphericity factor is inversely proportional to the spherical shape of the bead (Lee et al., 2013). This spherical shape is due to the high viscosity of the gum Arabic-sodium alginate solution. High-viscosity solutions are favourable for the formation of spherical beads. Although many polymer gels are used to entrap bacteria for use as agricultural inoculants, encapsulating bacteria in polymer matrix of alginate and gum Arabic is one of the most effective and safest ways to introduce microbial cells into the soil. Gum Arabic is a polysaccharide and glycoprotein-based hydrocolloid with high stability and potential water solubility. It forms a protective matrix around the core material (Lamsen et al., 2020). The rice bran provides structural support to the bead by increasing the viscosity of the polymer solution. The removal of water from the encapsulated bead causes the bead to collapse and lose its spherical shape (Chan et al., 2011). But the addition of rice bran as the filler provides structural support to the bead and protects it from collapse and shrinkage maintaining its sphericity. Similar results have been earlier reported in the study described by Chotiko and Sathivel (2016) that used starch as a filler (structural support). Furthermore, the addition of rice bran displays a spherical shape to the bead, enhances bacterial cell survival, and protects against heat stress (Raddatz et al., 2020; Tianwitawat and Klaiprasitti, 2023). Therefore, encapsulating microbial cells using rice bran, alginate and gum Arabic allows for better handling, longer viability, stress tolerance, good flowability, and less leaching out of microbial cells. The encapsulating matrix is also the primary reason. This corresponds to the excellent encapsulation efficiency (EE) of 94.11%. The high encapsulation efficiency was attributed to the high potential of gum Arabic to form complexes with sodium alginate. The addition of rice bran offers an additional possible benefit of reducing bacterial leakage. Although both gum Arabic and sodium alginate are negatively charged polysaccharides, the local positive charges in the protein fraction of gum Arabic enable its electrostatic interaction with negatively charged carboxylate groups in alginate at pH 4 and 7 (Sabet et al., 2021). This complex formation could be beneficial for pH-targeted delivery systems to soil (Sabet et al., 2020). The addition of rice bran helps to reduce bacterial loss during the encapsulation procedure and avoid bacterial leakages (Chotiko and Sathivel, 2016). The addition of rice bran in the encapsulation polysaccharide solution has earlier been shown to enhance the loading of *Lactobacillus* in the pectin capsules (Chotiko and Sathivel, 2016). These results were much higher than the previously reported 70.83% for encapsulating bacteria in starch and alginate matric materials (Rohman et al., 2021).

The formulations must display high microbial viability to exert positive plant growth-promoting effects when inoculated into the soil. Thus, the developed biofertilizer was studied for the survival of viable cell count in beads during storage (Figure 1). The viable cell count in the encapsulated bead decreased during the initial phase of storage (Figure 2A). This decrease in cell count can be due to the decrease in the moisture content or the death of unstable cells that do not become dormant during the drying process (Ivanova et al., 2006). Drying the beads decreases bacterial enzymatic activity and cell metabolism, which increases the shelf-life of biofertilizer (Puguan et al., 2015). Similar results were observed by Ivanova et al. (2006) who observed that in dried beads, after 7 days of storage at any conditions, the viable cell count remains constant. Therefore, drying the beads can be recognized as an efficient way of long-term storage (Berninger et al., 2018). Compared to the beads stored at room temperature, the beads stored at 4°C showed a higher survival rate (Figure 2B). Earlier reports also suggest that the storage of biofertilizers is generally claimed to be better under refrigeration than in room settings (Phiromtan et al., 2013; Liu et al., 2019). The higher survival rate of bacteria was observed in the dried beads as the bacteria converted itself to stable dormant structures providing better protection to the bacteria cells. Also, it is important to note that dried beads were free from contamination throughout the storage period, while the wet beads (both stored at room temperature and at 4°C) were prone to bacterial and fungal contamination over the long storage periods. Therefore, the air-dried beads stored at 4°C were favorable for bacterial survival over the long storage hours. The encapsulation protects the bacterial cells from contamination and the rice bran and gum Arabic which acts as a good carbohydrate source provides effective protection to the cells (He et al., 2015). Previous studies have also confirmed that the encapsulation of bacteria in alginate maintains the viability of biofertilizer for upto 3 months (Sultan et al., 2023).

The biodegradation of beads is an important aspect in view of sustainability. It is interesting to check the behavior of encapsulated beads in the soil to check biodegradability and bacterial release. The blending of alginate with gum Arabic and rice bran provides the bead impenetrable outer constitution with high mechanical strength, which prohibits the perforation of water into the bead. The mass of the bead decreases continuously which confirms their biodegradability behavior (He et al., 2015). The addition of the gum Arabic with alginate enhances the biodegradability of the beads. Our results are in correlation with the findings of Xie et al. (2012) and He et al. (2015) who have observed that the addition of other polysaccharides with sodium alginate enhances the biodegradability of the microcapsules.

**Figure 10:**
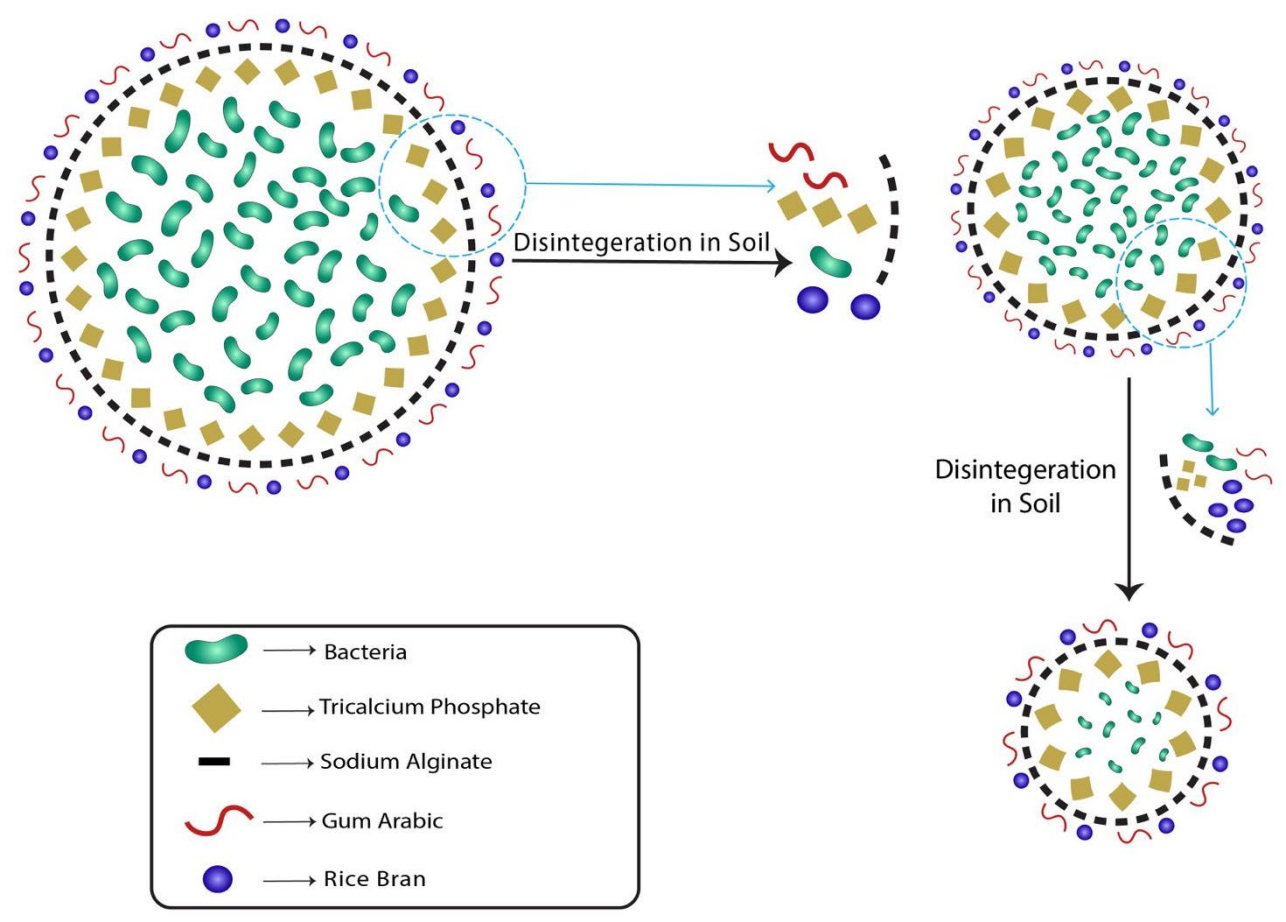
Mechanism of slow and gradual bacterial release from RB-GA beads through the dissolution of outer layer.

The cumulative bacterial release from the beads was studied to investigate its release kinetics. The release of bacterial cells from the bead was observed in two phases, initial burst followed by steady and continuous release (Figure 4). The bacterial cells were found to be released slowly in a gradual manner. The initial burst or rapid release of bacterial cells in the initial incubation periods was due to the fact that bacteria on the external surface of the bead and on the external outer surface were promptly liberated. The interaction of alginate and gum Arabic with rice bran provides a non-porous dense structure to the bead which prohibits the rapid diffusion of water into the core. Lower diffusion of water into the bead, results in smaller release channels. Therefore, in the later stages, the bacterial release was dependent on the dissolution of the outer layer into the saline solution that allowed the bacteria to diffuse out of the bead (Figure 10). Literature studies have reported a similar observation confirming that dissolution curves follow a similar pattern of initial burst followed by slow and gradual release (Liew et al., 2006).

The internal structure of bead as confirmed by XRD analysis and FTIR spectroscopy, confirmed the presence of the presence and authenticity of the tricalcium phosphate, gum Arabic, sodium alginate and rice bran. Due to its close molecular packing and crystallization, it exhibited a characteristic semi-crystalline structure. For β-tricalcium phosphate (TCP), the characteristic peak was witnessed at 29.149° has the estimated to d-space = 3.06104 Å, which was found to be similar as cited in the literature (Jana et al., 2015; Wahyudi et al., 2020). In the case of rice bran and gum acacia, the maximum intensity was obtained at 2θ = 19.895° (Sulamain and Elbasheer, 2016). Beads made using TCP, rice bran, and alginate exhibit signature peaks between 19–29°, indicating the semi-crystalline form of the microbeads (Cui et al., 2022).

The exterior of the bead was observed to be dense matrices. Earlier, Chitprasert et al. (2012) and Chotiko and Sathivel (2016) reported that due to the addition of rice bran, it was hard to find bacterial cells entangled in aluminum carboxymethyl cellulose capsules because rice bran provides more density to the beads. The high concentration of sodium alginate and gum Arabic aids in higher viscosity and creation of spheroid beads (Govindasamy et al., 2019). Chitprasert et al. (2012) also observed that the incorporation of rice bran in aluminum carboxymethyl cellulose microcapsules enhances the density of the beads that provide heat-protecting properties to the beads.

The supremacy of developing a sustainable bioformulation can only be achieved if it could show a visible effect on promoting plant growth and helping the plant to uptake essential macronutrients. The inoculant must survive in the soil, establish itself and promote the growth of crop (Verma et al., 2023). The significant differences in the growth of wheat seedlings can be attributed to the potent plant growth-promoting potential of *M. gitemensis* (Kaur and Kaur, 2021). In our previous study, we observed that inoculation of wheat seedlings with the pure liquid culture of *M. gitemensis* significantly provides growth augmentation in wheat. Nitrogen assimilation was not affected by the inoculation of beads which is due to the fact that *M. gitemensis* lack the nitrogen-fixing ability (Kaur and Kaur, 2021). Maximum nitrogen uptake was seen in positive control, which may be due to the presence of nitrogen-fixing rhizobacteria present in vermicompost. Different studies have reported wheat growth promotion as a consequence of liquid biofertilizer application (Kachroo and Razdan, 2006; Chitprasert and Sutaphanit, 2014; Singh et al., 2022) but the present study is the immediate report giving evidence about the plant growth enhancing potential of RB-GA encapsulated bacteria. The study provides evidence that RB-GA beads allow the slow release of bacteria into the soil over a longer time period which help the bacteria to survive and colonize the plant roots effectively. Compared to liquid biofertilizers, the RB-GA encapsulated bacteria have better potential for plant growth promotion.

## 5. Conclusion

The present study provides a sustainable route for the delivery of rhizobacteria to the soil through encapsulation in RB-GA beads. The addition of gum Arabic provides a stable structure to the bead due to its high potential of forming complexes with sodium alginate. The amalgamation of rice bran with gum Arabic and sodium alginate provides protection against bacterial leakage. The combination of these three components provides structural stability to the bead reducing bacterial loss by diffusion of water into the inner core. Thus, this bioformulation offers a sustainable slow, and continuous delivery system of the rhizobacteria into the soil. The dried RB-GA encapsulated *M. gitemensis* has shown a high survival rate over a storage period of 120 days. The mass of the bead was found to decrease continuously with incubation time which confirmed their biodegradability behavior. It took approximately 4 months for the bead to completely disintegrate into the soil confirming the slow-release behavior of the bead.

The plant growth-promoting ability of RB-GA encapsulated *M. gitamensis* was assessed by supplying the dried formulations to the pot-grown wheat seedlings allowed to grow in natural open field conditions. Compared to negative and positive controls, the germination rate, root length, shoot length, fresh weight, and dry weight of the seedlings were significantly higher in the RB-GA treated pots. The study provides evidence that RB-GA beads allow the slow release of bacteria into the soil over a longer time period which helps the bacteria to survive and colonize the plant roots effectively. Overall, the development of a biodegradable microencapsulated biofertilizer offers a promising solution for sustainable agriculture, providing a safe and effective alternative to traditional chemical fertilizers while promoting soil health and reducing environmental impact. Futuristic research demands work on testing the efficacy of encapsulated biofertilizers in the field conditions. Well-designed field trials should be conducted to bring these formulations from the lab to the field.

## Data availability statement

The raw data supporting the conclusions of this article will be made available by the authors, without undue reservation.

## Author contributions

RK: Conceptualization, Data curation, Investigation, Methodology, Project administration, Software, Writing – original draft. SK: Conceptualization, Data curation, Formal analysis, Investigation, Methodology, Supervision, Writing – review & editing. VD: Data curation, Formal analysis, Investigation, Writing – review & editing. CK: Writing – original draft, Writing – review and editing, Data analysis. NA: Data analysis, Resources, Writing – review and editing, Funding acquisition. AA: Writing – review and editing, Funding acquisition.

## Funding

The author(s) declare financial support was received for the research, authorship, and/or publication of this article.

## Conflict of interest

The authors declare that the research was conducted in the absence of any commercial or financial relationships that could be construed as a potential conflict of interest.

## Publisher’s note

All claims expressed in this article are solely those of the authors and do not necessarily represent those of their affiliated organizations, or those of the publisher, the editors and the reviewers. Any product that may be evaluated in this article, or claim that may be made by its manufacturer, is not guaranteed or endorsed by the publisher.

## Footnotes

^1^ https://www.pau.edu/content/extserv/fo/11.pdf
